# Physiologically Based Pharmacokinetic (PBPK) Modeling to Predict CYP3A-Mediated Drug Interaction between Saxagliptin and Nicardipine: Bridging Rat-to-Human Extrapolation

**DOI:** 10.3390/pharmaceutics16020280

**Published:** 2024-02-16

**Authors:** Jeong-Min Lee, Jin-Ha Yoon, Han-Joo Maeng, Yu Chul Kim

**Affiliations:** 1 Department of Digital Anti-Aging Healthcare, Inje University, Gimhae 50834, Republic of Korea; orolzi@naver.com; 2 College of Pharmacy, Gachon University, Incheon 21936, Republic of Korea; jinha89@daum.net; 3 Department of Pharmaceutical Engineering, Inje University, Gimhae 50834, Republic of Korea

**Keywords:** drug–drug interaction, CYP3A4, nicardipine, saxagliptin, PBPK modeling

## Abstract

The aim of this study was to predict the cytochrome P450 3A (CYP3A)-mediated drug–drug interactions (DDIs) between saxagliptin and nicardipine using a physiologically based pharmacokinetic (PBPK) model. Initially, in silico and in vitro parameters were gathered from experiments or the literature to construct PBPK models for each drug in rats. These models were integrated to predict the DDIs between saxagliptin, metabolized via CYP3A2, and nicardipine, exhibiting CYP3A inhibitory activity. The rat DDI PBPK model was completed by optimizing parameters using experimental rat plasma concentrations after co-administration of both drugs. Following co-administration in Sprague–Dawley rats, saxagliptin plasma concentration significantly increased, resulting in a 2.60-fold rise in AUC, accurately predicted by the rat PBPK model. Subsequently, the workflow of the rat PBPK model was applied to humans, creating a model capable of predicting DDIs between the two drugs in humans. Simulation from the human PBPK model indicated that nicardipine co-administration in humans resulted in a nearly unchanged AUC of saxagliptin, with an approximate 1.05-fold change, indicating no clinically significant changes and revealing a lack of direct translation of animal interaction results to humans. The animal-to-human PBPK model extrapolation used in this study could enhance the reliability of predicting drug interactions in clinical settings where DDI studies are challenging.

## 1. Introduction

Diabetes mellitus, a chronic metabolic disorder characterized by impaired insulin function or production, remains a significant global health concern. Its prevalence, particularly type 2 diabetes, is intricately linked to a spectrum of complications, notably hypertension and various cardiovascular diseases [1]. The management of diabetes extends beyond glycemic control to mitigating associated comorbidities such as hypertension and dyslipidemia, crucial risk factors for kidney disease, peripheral vascular ailments, and stroke [2]. The interplay of these conditions necessitates concurrent therapeutic approaches targeting both diabetes and its related cardiovascular risks.

In this context, pharmacological interventions often involve multiple medications, leading to concerns about potential drug–drug interactions (DDIs). The metabolism of many drugs occurs predominantly through hepatic cytochrome P450 (CYP450) enzymes, and their interactions have been extensively reported [3]. Specifically, the degree of dependency of drug metabolism on specific CYP450 enzymes significantly impacts the likelihood of interactions with certain inhibitors or inducers, altering plasma concentrations and subsequent pharmacological effects [4].

Saxagliptin, a competitive inhibitor of dipeptidyl peptidase-4 (DPP-4), stands as a cornerstone in diabetes management, enhancing insulin production by elevating glucagon-like peptide-1 (GLP-1) levels and promoting the conversion of GLP-1 into its inactive form [5]. This mechanism of action, dependent on glucose levels, offers a favorable profile by mitigating the risks of hypoglycemia and weight gain [5]. However, its metabolism, primarily via CYP3A4, with a higher metabolic activity compared to CYP3A5 [6], prompts considerations regarding potential interactions when co-administered with other medications impacting CYP3A4.

Nicardipine, a dihydropyridine derivative, functions by dilating blood vessels through the inhibition of calcium channels in vascular smooth muscle cells, effectively used in the treatment of hypertension, angina pectoris, and various cardiovascular conditions [7,8]. Nicardipine has been shown to be rapidly and extensively metabolized by the liver and is known to be a potent CYP3A4 inhibitor [9,10]. Its rapid metabolism, with no unchanged excretion in urine or bile, coupled with its role as a competitive CYP3A4 inhibitor, raises concerns about potential interactions when concurrently administered with saxagliptin. Specifically, it has been confirmed that DPP-4 inhibitors can enhance the effects of antihypertensive drugs such as calcium channel blockers. This suggests a potential improvement in therapeutic outcomes by combining DPP-4 inhibitors and calcium channel blockers, especially in patients with cardiovascular diseases. The co-administration of these two medications can be reasonably anticipated [11].

Therefore, this study aims to predict and evaluate potential CYP3A-mediated drug interactions between saxagliptin and nicardipine using physiologically based pharmacokinetic (PBPK) modeling technology [12]. PBPK modeling is an advanced computational approach used to predict the behavior of drugs in the body based on physiological parameters and drug-specific properties. Recent progress in PBPK modeling has led to more comprehensive and accurate predictions of drug interactions, aiding in drug development and clinical decision making. Key applications include assessing the impact of drug–drug interactions on pharmacokinetics and optimizing dosage regimens [13]. While previous PBPK model studies have attempted to predict drug interactions of saxagliptin associated with CYP3A4, it is crucial to note that these studies require actual clinical DDI results for modeling [14,15]. Our research involved validating a PBPK model using experimental data to predict drug interactions in rats. This model posits that saxagliptin’s metabolism in rats is primarily mediated by CYP3A2, an enzyme homologous to human CYP3A4 [16]. Subsequently, this model was extrapolated to a human PBPK model for the prediction of clinical drug interactions. The significance of this study lies in addressing the limitations of predicting clinical drug interactions based on animal studies through the PBPK model extrapolation approach, which incorporates various physiological and metabolic differences between the species.

## 2. Materials and Methods

### 2.1. Materials

Nicardipine hydrochloride (N0635, purity: >98.0%) and saxagliptin hydrate (A3435, purity: >98.0%) were purchased from Tokyo Chemical Industry (Tokyo, Japan). Midazolam, 1′-hydroxymidazolam (purity: >97.0%), and ketoconazole (purity: 99.0–101.0%) were kindly provided by Gachon University. Phenacetin (77440, purity: >98.0%), tolbutamide (T0891, purity: 97.0–103.0%), celecoxib (SML3031, purity: >98.0%),and β-Nicotinamide adenine dinucleotide 2′- phosphate reduced tetrasodium salt (NADPH) (N1630, purity: >93.0%) were purchased from Sigma-Aldrich (St. Louis, MO, USA). Human liver microsome (HLM), rat liver microsome (RLM), and recombinant rat CYP3A2 (rCYP3A2) were obtained from Xenotech (Lenexa, KS, USA). All other chemicals and solvents were of reagent or HPLC grade.

### 2.2. Saxagliptin Metabolic Stability

The intrinsic clearance (CL_int_) used in the PBPK model was determined from the elimination rate of saxagliptin by rCYP3A2. The mixtures of saxagliptin and rCYP3A2 were pre-incubated at 37 °C for 3 min. The final mixtures consisted of 0.1 M phosphate buffer (pH 7.4), 1 mM NADPH, 5 μM saxagliptin, and 10 pmol CYP/mL. After initiating the reaction by adding NADPH solution, the sampling was carried out at 37 °C for 0, 5, 10, 20, and 30 min in triplicate and stopped with methanol containing 200 ng/mL tolbutamide as an internal standard (IS) for HPLC analysis.

### 2.3. CYP Inhibition Assay by Nicardipine

The CYP inhibition assay was performed to obtain parameters for CYP3A inhibition of nicardipine used in the DDI PBPK model. The inhibitory effect of nicardipine on CYP3A was assessed using midazolam, a CYP3A4 probe substrate. We measured changes in the formation of 1′-hydroxymidazolam, the major metabolite of midazolam metabolized by CYP3A, in the presence of nicardipine. A pre-reaction mixture containing 0.1 M phosphate buffer (pH 7.4), HLM or RLM, nicardipine, and midazolam was pre-incubated at 37 °C for 3 min. The reaction was initiated by adding NADPH with final conditions of 0.5 mg microsomal protein/mL, 1 mM NADPH, 2.5 μM midazolam, and nine nicardipine concentrations ranging from 0 to 100 μM. After incubation at 37 °C for 5 min, the reaction was terminated by adding ice-cold acetonitrile containing 200 ng/mL phenacetin, an IS for liquid chromatography tandem mass spectrometry (LC-MS/MS) analysis. The extent of inhibition caused by nicardipine on the formation of 1′-hydroxymidazolam was quantified at each concentration tested. The IC_50_ value was determined through nonlinear regression analysis using GraphPad Prism software (San Diego, CA, USA) [17].

### 2.4. Non-Clinical Drug–Drug Interaction Study

#### 2.4.1. Animals

Male Sprague–Dawley (SD) rats, 7 weeks old (180–210 g), were purchased from Hana Biotech (Pyeongtaek, Republic of Korea). The rats were kept in a room with a relative humidity of 50–60%, a temperature of 21 ± 2 °C, and a 12 h light/dark cycle and were acclimatized to these conditions for a week. The protocol for these experiments was approved by Inje University’s Animal Care Committee (Inje 2022-012, Gimhae, Republic of Korea).

#### 2.4.2. Study Design

The rats were randomly divided into three groups: nicardipine alone (15 mg/kg), saxagliptin alone (5 mg/kg), and co-administration of nicardipine and saxagliptin (15 and 5 mg/kg, respectively). The drug was orally administered via a feeding tube, employing a total dosing volume of 5 mL/kg across all groups. Blood samples (approximately 200 μL) were collected from the jugular vein at 0.25, 0.5, 1, 2, 3, 4, 6, 8, 12, and 24 h post-dose. The timing of blood collection was determined based on previous studies, aiming to capture appropriate pharmacokinetic profiles, including the time to reach the maximum concentration (T_max_) of saxagliptin and nicardipine in rats [18,19]. The blood samples were promptly centrifuged at 15,000 rpm for 5 min at 4 °C. The resulting plasma was stored at -70 °C until HPLC or LC-MS/MS analysis.

#### 2.4.3. Pharmacokinetic Analysis

Noncompartmental analyses of nicardipine and saxagliptin were performed using PK-solver ver 2.0 (Center for Instrumental Analysis, Pharmaceutical University, Nanjing, China), an add-in program for Microsoft Excel [20]. The calculated pharmacokinetic parameters included the following: area under the time–plasma concentration curve (AUC) between from0 to infinity (AUC_inf_); maximum plasma concentration (C_max_); time to reach maximum concentration (T_max_); terminal half-life (t_1/2_); oral clearance (CL/F). Pharmacokinetic parameters are expressed as the mean ± standard deviation (SD). For statistical analysis, a Student’s *t*-test was performed using GraphPad Prism software (San Diego, CA, USA) with a significance threshold of less than 0.05.

### 2.5. LC-MS/MS and HPLC Analysis

Saxagliptin concentration levels for plasma and microsomal metabolism samples were quantified by the LC-MS/MS system. The LC-MS/MS system consisted of an Agilent 6490 triple quadrupole mass spectroscopy system and a 1290 infinity HPLC system (Agilent Technologies, Santa Clara, CA, USA). The separation of compounds was accomplished by a reversed-phase column (Synergi Polar-RP 80 Å, 150 × 2.0 mm, 4 μm, Phenomenex, Torrance, CA, USA) with a security guard. The mobile phase for analyzing saxagliptin and phenacetin (internal standard, IS) was a mixture of MeOH and 0.1% formic acid in water (70:30, *v*/*v*), and the flow rate was 0.2 mL/min in an isocratic condition. Multiple reaction monitoring (MRM) modes were used for the quantitative study of the compound’s precursor ion and product ion transitions. In the positive electrospray ionization mode, the *m*/*z* values of saxagliptin and phenacetin (IS) were 316.2 → 180.2 and 180.0 → 162.2, respectively. In addition, 1′-hydroxy midazolam was analyzed under the following conditions. The mobile phase was a mixture of 0.1% formic acid in water and ACN (50:50 *v*/*v*), with a flow rate of 0.2 mL/min. The column temperature was kept constant at 25 °C. The following ion values (*m*/*z*) were obtained by monitoring the transitions: 1′-hydroxy midazolam, 342.0 → 203.1; phenacetin (IS), 180.0 → 162.2. The data analysis was achieved using MassHunter software version A.06.00 (Agilent Technologies, Santa Clara, CA, USA).

The plasma concentration of nicardipine was quantified using HPLC. The HPLC system included a degasser pump (L-2130), a column oven (L-2300), an autosampler (L-2200), and a diode array detector (L-2455). The analysis was carried out using the software Hitachi LaChrom D-2000 Elite (Merck-Hitachi, Tokyo, Japan). The separation was accomplished using a reversed-phase column (Synchronis C18 150 × 4.6 mm, 5 μm, Thermo Scientific, Waltham, MA, USA) with a guard column (security guard C18 4 × 2.0 mm, Phenomenex, Torrance, CA, USA). The mobile phase was comprised of phases A (ACN) and B (0.015 M potassium phosphate monobasic (KH_2_PO_4_) with 2.8 mM triethylamine in water) [18]. The mobile phase was A:B = 60:40, with a flow rate of 1.5 mL/min. The temperature of the column oven controller was maintained at 40 °C, and 50 μL of sample was injected into the system [18]. Nicardipine and celecoxib (an IS) were detected at 254 nm [18].

### 2.6. PBPK Model Construction and Simulation

The PBPK models for saxagliptin, nicardipine, and their DDIs were developed using the PK-Sim program (version 11, Open Systems Pharmacology Suite, Germany) [21]. The overall PBPK modeling scheme was depicted in a simplified manner in Figure S1. The whole-body PBPK model framework set as the default in PK-Sim was used, which is permeability-limited for both rat and human models. The PK-Sim standard distribution model assumes 4 sub-compartments per organ, i.e., compartments for blood cells, plasma, interstitial space, and cellular space. All organs and physiological parameters comprising the models are shown in Table S1. Partition coefficients between organs and plasma and cellular permeability were calculated using the PK-Sim method [22].

Organ-specific protein concentrations were determined through a composite of reference concentrations and the overall organ expression levels. For human models, the gene expression repository within PK-Sim was employed to represent the enzyme and transporter expression levels across various organs, utilizing protein gene expression data derived from previously published RT-PCR experiments [23,24]. Rat-specific organ protein expression levels were acquired from the Bgee database [25] with rat liver microsomal protein levels of 44.8 mg protein/g liver and CYP3A2 levels at 101 pmol CYP/mg protein [26,27].

Regarding CYP inhibition in the PBPK models, given the known competitive inhibition of CYP3A4 by nicardipine [10], the following equation integrated into PK-Sim was employed.

(1) $${CL}_{int,app}={CL}_{int}\frac{1}{1+\frac{\left[I\right]}{K_{i}}}$$

where CL_int,app_ denotes the apparent intrinsic clearance, K_i_ represents the inhibitory constant, and [I] represents the concentration of the free inhibitor. We assumed first-order metabolism of saxagliptin by CYP3A, with the specific clearance being adjusted according to Equation (1). To account for DDIs in the human model, the K_i_ value for CYP3A4/5 of nicardipine was sourced from the literature and integrated into the model. For the rat model, the rat K_i_ of nicardipine was estimated using the ratio between the human experimental IC_50_ and the literature K_i_ values.

The other physicochemical and pharmacokinetic parameters for the PBPK model were obtained from the literature or experiments, and, if needed, refined by fitting to plasma concentration data. These parameters include lipophilicity (logP or logD), solubility, log acid dissociation constant (pK_a_), fraction unbound (f_u_), permeability, intrinsic clearance, and others (Table 1 and Table 2).

The PBPK modeling strategy begins with constructing PBPK models for each saxagliptin and nicardipine, with saxagliptin’s metabolism specifically set to occur via CYP3A2 in rast. These models were subsequently optimized by fitting with rat plasma concentration profiles after intravenous or oral administration. Plasma concentration data for saxagliptin and nicardipine in the literature were extracted using Plot Digitizer (version 2.6.9, http://plotdigitizer.sourceforge.net/, accessed on 1 July 2023). Further optimization involved developing a DDIs model for co-administration of saxagliptin and nicardipine, achieved by refining the model against saxagliptin plasma concentrations in rats with post-concurrent administration. In the rat DDI PBPK model, the obtained K_i_ value of nicardipine, as mentioned earlier, was utilized.

After finalizing the rat PBPK model, a similar methodology was employed to build the human PBPK model. The physicochemical and pharmacokinetic parameters primarily relied on values derived from the completed rat PBPK model, with metabolism configured via CYP3A4/5. Plasma concentration data for saxagliptin and nicardipine in humans were obtained from the literature and utilized to refine the model parameters in human PBPK models. The human demographic data for the PBPK model was also input from the literature sources that provided plasma concentration. For the human DDI PBPK model, nicardipine’s K_i_ on CYP3A4, obtained from the literature, was integrated into the model. Finally, this completed model simulated the concurrent plasma concentrations of both drugs at clinical doses.

Additionally, the Monte Carlo approach was used to compare observed data with projected models, assuming a lognormal distribution using the mean and variance reported in the literature for parameter uncertainties [37].

### 2.7. Sensitivity Analysis

A sensitivity analysis was conducted to assess the impact of model parameters on the C_max_ and AUC_inf_ of saxagliptin in rat and human DDI PBPK models. The examined model parameters comprised optimized values as well as those anticipated to exert a noteworthy influence on C_max_ and AUC_inf_. The sensitivity coefficient (SC) is calculated using Equation (2).

(2) $$SC=\frac{\Delta PK}{PK}÷\frac{\Delta p}{p}$$

where ∆*PK* represents the change in predicted C_max_ and AUC_inf_, with *PK* representing the initial value of C_max_ and AUC_inf_. *∆p* represents the alteration of the assessed model parameters, with *p* representing the initial value of these parameters. A SC value of +1.0 indicates that a +10% change in the model parameter causes a +10% change in the C_max_ and AUC_inf_.

### 2.8. Evaluation of PBPK Model

The PBPK model underwent evaluation through a visual comparison between predicted plasma concentration profiles and observed data. The visual assessment involved juxtaposing the predicted concentration with a 95% prediction interval against empirical data sourced from the literature or in-house experiments. Additionally, goodness-of-fit (GOF) plots were employed to compare predicted and observed values for plasma concentrations, C_max_, and AUC_inf_. Predictions were deemed successful if they did not deviate more than 2-fold from observed values.

Quantitative evaluation encompassed the computation of mean relative deviation (MRD) for individual concentrations and the determination of the geometric mean fold error (GMFE) for derived pharmacokinetic parameters [38]. MRD, calculated using Equation (3), utilized ‘C_obs_’ for observed plasma concentrations, ‘C_pred_’ for predicted values, and ‘N’ denoting the total count of observed values. Simultaneously, GMFE, computed according to Equation (4), incorporated ‘n’ as the number of studies considered in the analysis. MRD and GMFE values ≤2 were considered adequate model performance metrics.


(3) $$MRD=10^{x};\;x=\sqrt{\frac{\sum_{i=1}^{N}{\left({log}_{10}C_{obs}-{log}_{10}C_{pred}\right)}^{2}}{N}}$$

(4) $$GMFE=10^{\sum \left|{log}_{10}(\frac{predicted\;PK\;parameter}{observed\;PK\;parameter})\right|/n}$$

The evaluation of the predictive capacity of DDI modeling centered on the assessment of DDI AUC_inf_ and DDI C_max_ ratios, computed through Equations (5) and (6), respectively. The determination of the GMFE for each DDI parameter ratio was conducted utilizing Equation (3). This equation served as a pivotal tool in quantifying the predictive accuracy of the model for DDI-related parameters.


(5) $$DDI\;{AUC}_{inf\;\;}ratio=\frac{{AUC}_{inf,\;victim\;drug\;co-administration}}{{AUC}_{inf,\;victim\;drug\;single\;administration}}$$

(6) $$DDI\;C_{max\;\;}ratio=\frac{C_{max,\;victim\;drug\;co-administration}}{C_{max,\;victim\;drug\;single\;administration}}$$

## 3. Results

### 3.1. Metabolic Stability of Saxagliptin

To integrate into the rat PBPK model, the CL_int_ of saxagliptin was determined through a metabolic stability study using rCYP3A2, given that saxagliptin is predominantly metabolized by CYP3A4/5. After incubation with the rCYP3A2 reaction mixture, saxagliptin exhibited a half-life of 34.8 min, yielding a calculated CL_int_ of 1.99 μL/min/pmol CYP, which was applied in the PBPK model (Table 1).

For the human PBPK model, CL_int_ from CYP3A4 and CYP3A5 were obtained from the literature and utilized (Table 1) [6].

### 3.2. Determination of CYP450 Inhibition Potential of Nicardipine

**Figure 1:**
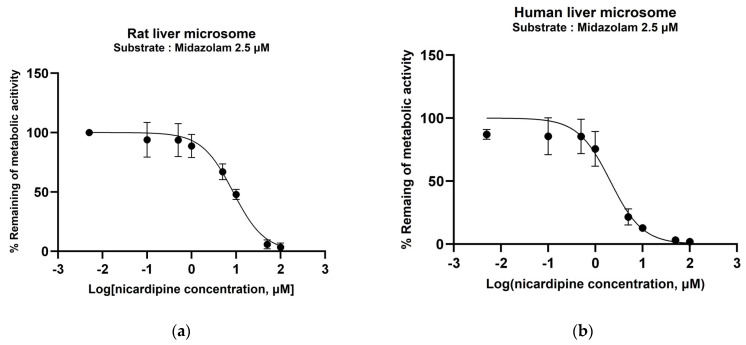
Remaining percentage (%) of midazolam metabolic activity at various concentrations of nicardipine in rat (**a**) and human (**b**) liver microsomes. Circle symbols represent observed values (mean ± standard deviation), while the black line represents a fitted line obtained through nonlinear regression.

IC_50_ of nicardipine for rat CYP3A2 or human CYP3A4/5 was determined by measuring the metabolic inhibition of midazolam, the CYP3A probe drug, in rat and human liver microsomes. Nonlinear regression was employed, yielding IC_50_ values of 8.59 (*R*^2^ = 0.9547) and 2.08 (*R*^2^ = 0.9265) μM for rat and human microsomes, respectively (Figure 1).

### 3.3. In Vivo Pharmacokinetic Studies

**Figure 2:**
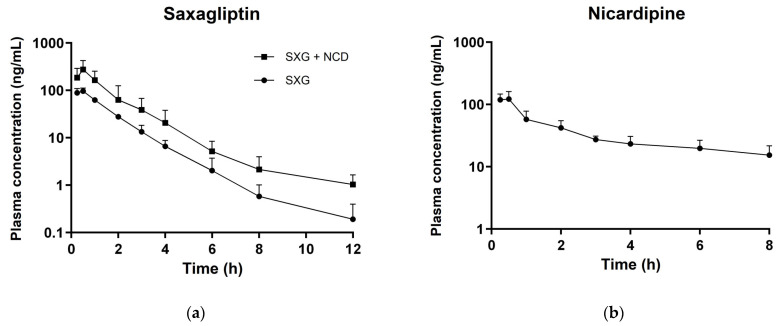
Plasma concentration of saxagliptin in rats after oral administration of saxagliptin alone (5 mg/kg) and co-administration of saxagliptin (5 mg/kg) with nicardipine (15 mg/kg) (**a**); Plasma concentration of nicardipine in rats after oral administration (15 mg/kg) (**b**). Circle symbols represent single administrations, while square symbols denote combination administrations. SXG, saxagliptin; NCD, Nicardipine.

The plasma concentration–time curves and PK parameters for oral administration of 5 mg/kg saxagliptin, alone and in combination with 15 mg/kg nicardipine in rats, were presented in Figure 2a and Table 3, respectively. When administered alone, saxagliptin exhibited a C_max_ of 98.5 ± 15.1 ng/mL, which increased to 280 ± 147 ng/mL when co-administered with nicardipine, representing a 2.84-fold increase. Furthermore, the co-administration resulted in an AUC_inf_ of 408 ± 213 ng·h/mL, representing a 2.61-fold escalation from the single administration’s value of 156 ± 22.9 ng·h/mL without nicardipine. These results suggest the potential for increased systemic exposure, leading to the manifestation of undesired toxicity, with concurrent drug administration in cardiovascular conditions that may accompany diseases such as diabetes and hypertension. As previously mentioned, nicardipine serves as a potent inhibitor of CYP3A, and saxagliptin is predominantly metabolized by CYP3A. Therefore, during co-administration, it is postulated that CYP3A inhibition by nicardipine contributes to a reduced metabolism of saxagliptin, leading to an increased systemic exposure of saxagliptin.

Meanwhile, the plasma concentration–time profile of nicardipine, administered at 15 mg/kg alone, was obtained for the construction of a rat PBPK model of nicardipine (Figure 2b), with corresponding PK parameters presented in Table 4. The C_max_ of nicardipine was 132 ± 35.2 ng/mL, the AUC_inf_ was 408 ± 121 ng·h/mL, and the t_1/2_ was 5.76 ± 1.45 h.

### 3.4. PBPK Model Construction

The physicochemical and pharmacokinetic parameters employed in the PBPK model for saxagliptin and nicardipine were obtained from the literature or in-house experimental data, as detailed in Table 1 and Table 2. Initially, parameters underwent optimization in the rat PBPK model, and those parameters that did not differ between rats and humans were retained in the human PBPK models. Specifically, for saxagliptin, the optimization of lipophilicity and specific intestinal permeability was conducted in a rat model and applied consistently to both rat and human models. Meanwhile, within the human models for both saxagliptin and nicardipine, dissolution shape and dissolution time were optimized considering tablet formulations.

### 3.5. Evaluation of PBPK Model

**Figure 3:**
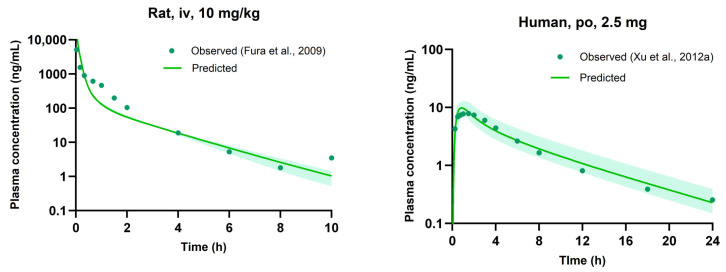

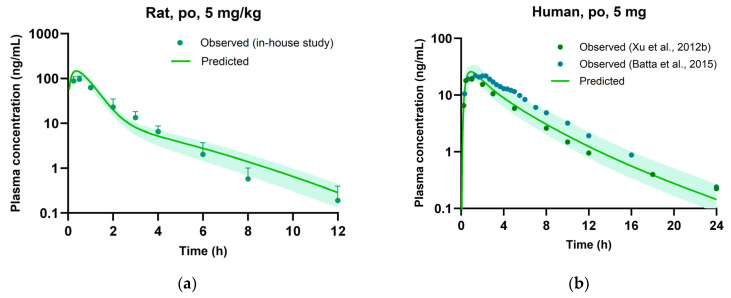
Plasma concentration-time profiles of saxagliptin in rats (**a**) and humans (**b**). The observed values from the literature and experiments are shown by circle symbols [19,39,40,41], while the predicted values of the models are represented by a solid line. The shaded region denotes the 95% prediction interval. SXG: saxagliptin, iv: intravenous, po: peroral.

Predicted PK parameters obtained from the PBPK model of saxagliptin were compared with observed values from a representative PK study in Table 5. In the case of rats, in-house experimental data were utilized, while for humans, the plasma concentrations following oral administration of 2.5 mg were used [39]. The predicted values compared to the observed values for the parameters exhibited a favorable predictive capacity within a two-fold range. Figure 3 illustrates the plasma concentrations of saxagliptin predicted from the PBPK model at various doses, accompanied by a 95% prediction interval [19,39,40,41]. A comparison was made with both in-house experimental data and literature observations, all of which fell within the acceptable range.

**Figure 4:**
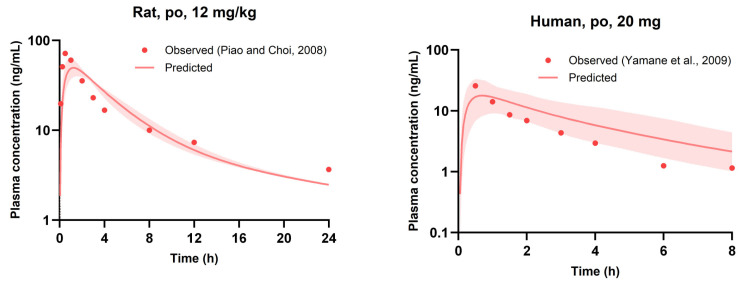

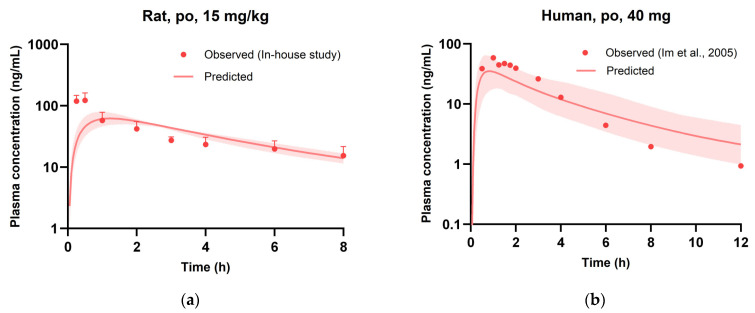
Plasma concentration-time profiles of nicardipine in rats (**a**) and humans (**b**). The observed values from the literature and experiments are shown by circle symbols [18,42,43], while the anticipated values of the models are represented by a solid line. The shaded region denotes the 95% prediction interval. NCD: Nicardipine, po: peroral.

Similar to the approach for saxagliptin, the PBPK model-predicted PK parameters and plasma concentrations for nicardipine, referred to as the perpetrator drug, were compared with observed values in Table 6 and Figure 4 [18,42,43]. As a result, nicardipine exhibited favorable predictive accuracy across various doses in both rat and human models, maintaining a predicted-to-observed parameter ratio within a 2-fold range. Moreover, across all dosages, observed concentrations of nicardipine consistently fell within the 95% prediction interval of the predicted concentration.

**Figure 5:**
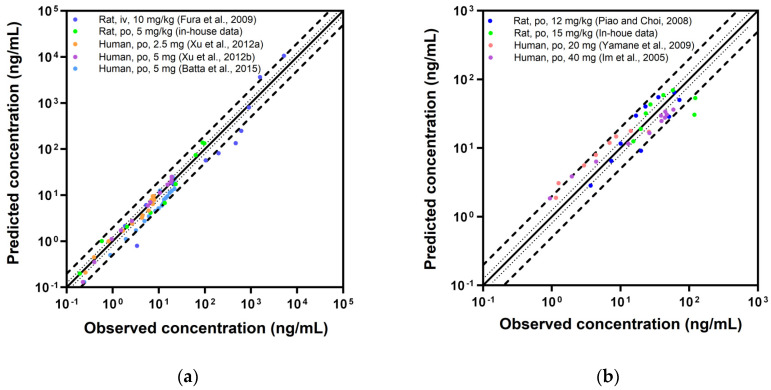

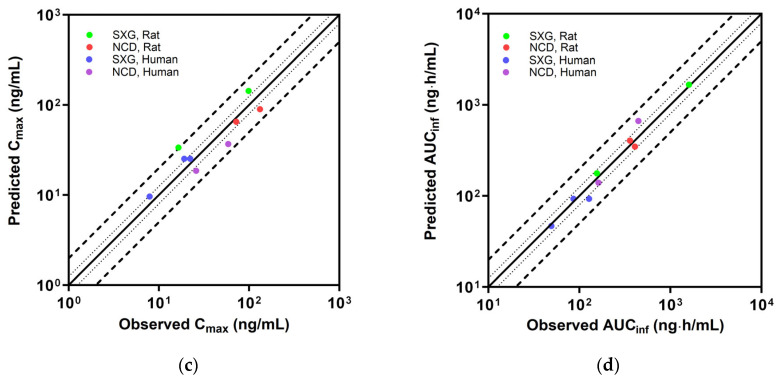
The goodness-of-fit plots, comparing observed plasma concentrations and PBPK model-predicted values for saxagliptin [19,39,40,41] (**a**) and nicardipine [18,42,43] (**b**), alongside the comparison of C_max_ (**c**) and AUC_inf_ (**d**) for both drugs. The solid line denotes the line of identity, while the dotted lines represent 1.25-fold deviation and the dashed lines represent 2-fold deviation.

The predictive accuracy of the PBPK models was illustrated using GOF plots for plasma concentration and exposure parameters, specifically C_max_ and AUC_inf_ (Figure 5). Additionally, quantitative evaluation was also conducted using MRD and GMFE, as detailed in Table 7. For saxagliptin, the MRD values ranged between 1.14 and 1.92 for the rat PBPK model and 1.17 and 1.60 for the human PBPK model. In the case of nicardipine, the MRD ranged from 1.69 to 2.08 for the rat model and 1.66 to 1.92 for the human model, respectively. With the exception of a single instance (nicardipine po 15 mg/kg in rats), all MRD values demonstrated an appropriate model predictability for plasma concentrations by meeting the predetermined criteria of being below 2. Furthermore, all GMFE_Cmax_ and GMFE_AUCinf_ values for both saxagliptin and nicardipine were also below 2, indicating robust predictive performance for exposure parameters (Table 7).

### 3.6. PBPK Modeling for DDIs

**Figure 6:**
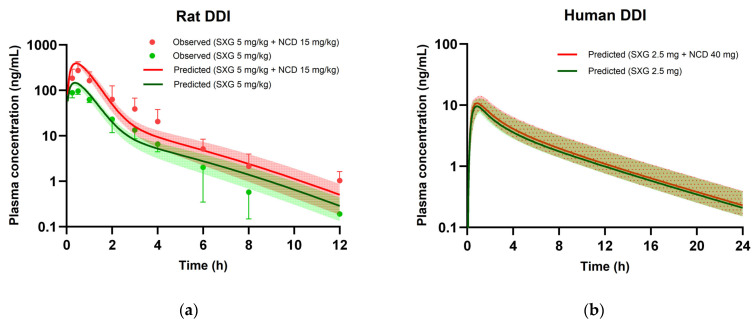
PBPK model simulations of saxagliptin plasma concentration-time profiles following oral administration without or with nicardipine. In rats (**a**), observed values are indicated by data points for groups treated with saxagliptin alone (green) and co-administered with nicardipine (red). Solid lines and shaded regions denote the mean and 95% prediction interval of predicted saxagliptin plasma concentration, respectively. In humans (**b**), solid lines represent the mean of predicted saxagliptin plasma concentration, while shaded (or dotted) regions represent the 95% prediction interval of predicted saxagliptin plasma concentration. SXG: saxagliptin, NCD: nicardipine.

Figure 6a presents the comparison between predicted plasma concentrations obtained from the rat DDI PBPK model and experimental values. Concurrently, Table 8 outlines the corresponding C_max_, AUC_inf_ values extracted from these plasma concentrations, and the resultant DDI ratio. The rat PBPK model effectively anticipated the increase in saxagliptin plasma levels at an oral dose of 5 mg/kg when co-administered with nicardipine at 15 mg/kg. In the co-administration study in rats, the C_max_ and AUC for saxagliptin increased by factors of 2.85 and 2.60, respectively, whereas the PBPK model predicted increments of 2.69 and 2.54, aligning closely with the experimental findings.

However, utilizing the same modeling approach in the human PBPK model indicated a less significant risk of DDI. When co-administered at clinical oral doses, 2.5 mg and 40 mg for saxagliptin and nicardipine, respectively, the human PBPK model predicted only a 1.10-fold increase in C_max_ and a 1.05-fold increase in AUC for saxagliptin, suggesting a lower potential for DDI in humans (Figure 6b and Table 8).

### 3.7. Sensitivity Analysis of DDI PBPK Models

**Figure 7:**
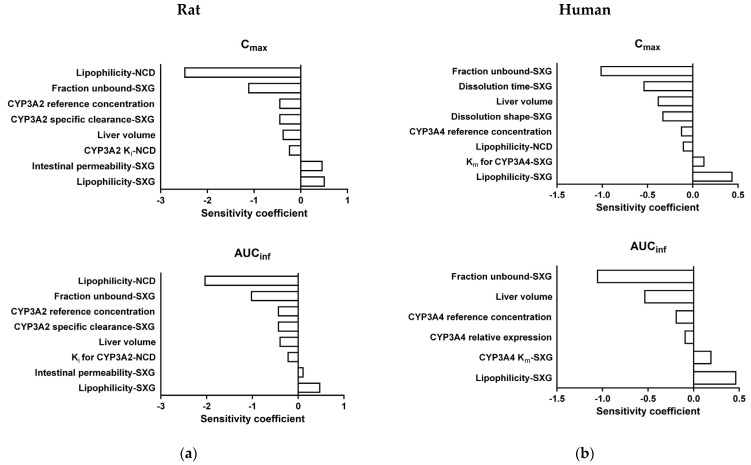
Sensitivity analysis of the DDI PBPK models in rats (**a**) and humans (**b**). The sensitivity coefficient of the parameters was estimated after a 10% variation in the parameters on C_max_ and AUC_inf_ of saxagliptin, a victim drug of drug–drug interaction, following co-administration of saxagliptin and nicardipine. SXG: Saxagliptin, NCD: Nicardipine.

A sensitivity analysis was conducted on PBPK models for DDI involving saxagliptin and nicardipine, aiming to assess the impact of various parameters on the models. Parameters with sensitivity coefficients exceeding 0.1 were considered sensitive, and their effects were depicted in Figure 7. In the rat model, nicardipine’s lipophilicity emerged as the most sensitive parameter for both the C_max_ and AUC_inf_ of saxagliptin. Additionally, the fraction unbound and lipophilicity of saxagliptin were identified as sensitive parameters, along with the CYP3A2 concentration and clearance associated with saxagliptin metabolism. The sensitivity of nicardipine’s CYP3A2 K_i_, related to drug–drug interaction activity, was relatively weak. In the human model, the unbound fraction of saxagliptin was found to be the most sensitive parameter for both C_max_ and AUC_inf_. Additionally, dissolution-related parameters, including dissolution time, dissolution shape, and lipophilicity of saxagliptin, were identified as sensitive factors influencing the saxagliptin exposures. Similarly to the rat model, parameters associated with CYP3A4 concentration or expression related to metabolism were found to be sensitive in the human model. However, unlike in the rat model, the nicardipine’s CYP3A4 K_i_ was not deemed sensitive.

## 4. Discussion

Drug interactions, particularly those mediated by CYPP450 isozymes, can significantly influence plasma concentrations, potentially impacting the effectiveness and safety of medications. In patients with chronic cardiovascular conditions, the co-administration of antihypertensive and antidiabetic drugs is common. Therefore, detecting potential alterations in drug concentrations due to CYP450-mediated interactions is crucial for optimizing treatment. Since conducting clinical DDI studies is generally challenging, predictions often rely on animal experiments. However, these predictions are limited by differences in physiology and pharmacokinetics between animals, and humans. To overcome these limitations and enhance predictivity, PBPK models have emerged as a valuable tool for DDI predictions, receiving endorsement from regulatory agencies such as the U.S. Food and Drug Administration (USFDA) [44]. Given the challenges of directly applying PBPK models in humans, this study initially constructed and validated PBPK models in rats, where in vivo experimental results are more feasible. Subsequently, these models were extrapolated to humans for predicting clinical DDIs.

Nicardipine, recognized for its potential to inhibit various CYPs (CYP3A4, CYP2C9, CYP2C19, and CYP2B1) [10], may increase plasma concentrations of drugs metabolized by affected CYPs, potentially leading to toxicity. Saxagliptin, an antidiabetic, is primarily metabolized by CYP3A4/5 and might be co-administered with the antihypertensive nicardipine in patients with cardiovascular chronic conditions. Human CYP3A4 shares about 70% homology with rat CYP3A2 [16]. Thus, assuming saxagliptin is primarily metabolized by CYP3A2 in rats, we constructed and validated a rat PBPK model using in vivo experiments after administering saxagliptin and nicardipine concurrently in rats. The plasma concentration of saxagliptin increased by more than 2-fold in vivo in rats, probably due to inhibition of rat CYP3A2 by nicardipine. We incorporated nicardipine’s CYP3A2 inhibition activity, obtained from rCYP3A2-based CYP inhibition experiments, into the rat PBPK model. This rat DDI model successfully simulated the elevation of saxagliptin plasma concentrations following oral co-administration of 5 mg/kg with nicardipine at 15 mg/kg in rats. In the animal experiment, saxagliptin’s C_max_ and AUC rose by factors of 2.85 and 2.60, respectively, while the model projected increments of 2.69 and 2.54 for C_max_ and AUC, respectively, demonstrating precise predictivity.

With the assumption that extrapolating a completed rat PBPK model to a human PBPK model using the same approach would improve the prediction accuracy of DDIs, we created a human PBPK model to predict DDIs at clinical drug doses. However, the predicted increase in saxagliptin’s plasma concentration (C_max_: 1.10-fold, AUC: 1.05-fold) with nicardipine co-administration did not reach a clinically significant threshold of 1.25-fold change in humans, unlike in rats. Several factors might account for this difference between rats and humans. Firstly, the simulated clinical dose of 40 mg for nicardipine resulted in a C_max_ of 36.4 ng/mL in humans, considerably lower than the rat C_max_ of 69.3 ng/mL. Moreover, the unbound fraction in plasma was 0.084 for rats and 0.01 for humans, indicating a lower unbound concentration involved in actual inhibition in humans. However, this approach has limitations in explaining the DDI differences between rats and humans because the calculated 1+[I]/K_i_ from the static model to predict DDI resulted in values of 1.03 for rats and 1.01 for humans, suggesting no significant difference [45,46].

On the other hand, differences in metabolic pathways between the rat and human PBPK models may contribute to varying predictions for DDI outcomes. In the rat model, saxagliptin metabolism was assumed to occur primarily via CYP3A2, while in the human model, it was assumed to involve both CYP3A4 and CYP3A5. When considering nicardipine as the CYP3A inhibitor in the PBPK models, it was set to inhibit CYP3A2 in rats and CYP3A4 in humans. This suggests that the unimpeded metabolism of CYP3A5 in humans resulted in a less dramatic inhibitory effect of nicardipine metabolism. 

Upon analysis of the sensitivity results, it became evident that not only metabolism-related parameters but also physicochemical properties-related parameters, such as lipophilicity and dissolution time, influence saxagliptin plasma concentration. Consequently, it is presumed that differences in the formulation or absorption processes of the two drugs in rats and humans could have impacted the divergent outcomes of drug interactions between species.

Several hypotheses have been previously mentioned to account for the differences in rat–human DDI prediction outcomes. However, to validate the predictability of clinical DDI, clinical drug interaction studies are necessary. On another note, predicting drug interactions in steady-state conditions after repeated administration is crucial, particularly when administering saxagliptin and nicardipine to patients with chronic conditions. PBPK model simulations under steady-state conditions using clinical dosage regimens yielded minimal changes in saxagliptin plasma concentrations, similar to the results of single-dose simulations. This study primarily aimed to extend the DDI PBPK model developed for rats post-single-dose administration to a human model, indicating limitations in simulating repeated dosing scenarios. Future research involving data on drug interactions in steady-state conditions after repeated administration in rats and model optimization may lead to the development of a human PBPK model that more accurately reflects real clinical situations.

## 5. Conclusions

We successfully developed a PBPK model to predict the DDIs between saxagliptin and nicardipine by extending the PBPK model from rats to humans. Although the approach has limitations in addressing all physiological and pharmacokinetic differences between rats and humans, the integration of validated PBPK models derived from animal experiments into human models holds promise for improving the accuracy of predicting clinical DDIs.

## Figures and Tables

**Table 1:** Physicochemical and pharmacokinetic parameters of saxagliptin for PBPK model.

Parameter		Unit	Rat		Human		Reference/ Comment
			Reference	Input	Reference	Input	
Molecular weight		g/mol	315.41				
Lipophilicity		logD_7.4_	−0.35	−0.7	−0.35	−0.7	[28]/Optimization
pK_a_ (Compound type)			7.9 (base)				[29]
Solubility (ref-pH)		mg/mL	17.6 (pH 7)				[30]
Fraction unbound			0.82	0.82	1	1	[19]
Specific intestinal permeability		10^−6^ cm/s	2.73	1.7	2.73	1.7	[31]/Optimization
Partition coefficient			Table S2				PK-Sim standard
Cellular permeability		10^−6^ cm/s		13		13	PK-Sim standard
	rCYP3A2 hCYP3A4 hCYP3A5	μL/min/pmol CYP	1.99	1.99			In-house data
CL_int_					0.38	0.38	[6]
					0.09	0.09	[6]
Renal clearance		mL/min/kg	38	38	1.9	1.9	[19]
Dissolution shape (tablet 2.5 mg)						0.68	Optimization
Dissolution time (tablet 2.5 mg)		min				120.5	Optimization
Dissolution shape (tablet 5 mg)						0.8	Optimization
Dissolution time (tablet 5 mg)		min				71.0	Optimization

**Table 2:** Physicochemical and pharmacokinetic parameters of nicardipine for PBPK model.

Parameter		Unit	Rat		Human		Reference/ Comment
			Reference Value	Input Value	Reference Value	Input Value	
Molecular weight		g/mol	479.59				
Lipophilicity		logD_7.4_	4.6				[32]
pK_a_ (Compound type)			8.1 (base)				[33]
Solubility (ref-pH)		mg/mL	7.9 (pH 7)				[34]
Fraction unbound			0.084	0.084	0.01	0.01	[9,35]
Specific intestinal permeability		10^−6^ cm/s	1.15				[36]
Partition coefficient			Table S2				PK-Sim standard
Cellular permeability		cm/s		0.09		0.09	PK-Sim standard
Total hepatic clearance	t_1/2_	min	0.62	0.62	4.5	4.5	[26]
K_i_	CYP3A2	μM	0.39	0.39			In-house data
	CYP3A4				0.06	0.06	[10]
Dissolution shape (tablet)						1.18	Optimization
Dissolution time (tablet)		min				5.59	Optimization

**Table 3:** Pharmacokinetic parameters of saxagliptin following oral single dosage of 5 mg/kg and co-administered with nicardipine 15 mg/kg to rats.

Group		SXG 5 mg/kg (*n* = 6)	SXG 5 mg/kg + NCD 15 mg/kg (*n* = 7)	
Parameter	Unit	Value		DDI Ratio
C_max_	ng/mL	98.5 ± 15.1	281 ± 147 *	2.85
T_max_	h	0.417 ± 0.129	0.464 ± 0.094	
t_1/2_	h	1.82 ± 0.737	2.97 ± 1.23	1.63
AUC_inf_	ng·h/mL	157 ± 23.1	408 ± 213 *	2.60
CL/F	L/h/kg	32.5 ± 5.15	14.3 ± 4.86 **	0.44

Data values are expressed as the mean ± standard deviation. Significance levels are denoted as * for *p* < 0.05 and ** for *p* < 0.01 compared to saxagliptin alone, determined by *t*-test analysis. SXG: saxagliptin; NCD: nicardipine.

**Table 4:** Nicardipine pharmacokinetic parameter, mean (± standard deviation), following oral administration to rats at 15 mg/kg (*n* = 7).

Parameter	Unit	Value
C_max_	ng/mL	132 ± 35.3
T_max_	h	0.357 ± 0.134
t_1/2_	h	5.76 ± 1.45
AUC_inf_	ng·h/mL	408 ± 121
CL/F	L/h/kg	39.7 ± 11.9

**Table 5:** Representative observed and predicted pharmacokinetic parameters of saxagliptin in rats and humans.

Parameter		Rat po 5 mg/kg			Human po 2.5 mg		
		Observed	Predicted	Predicted/ Observed	Observed [39]	Predicted	Predicted/ Observed
C_max_	ng/mL	98.5 ± 15.1	144	1.46	7.89	9.64	1.22
t_1/2_	h	1.82 ± 0.737	1.63	0.90	7.21	5.33	0.74
AUC_inf_	ng·h/mL	157 ± 23.1	177	1.13	49.1	46.9	0.96

**Table 6:** Representative observed and predicted pharmacokinetic parameters of nicardipine in rats and humans.

Parameter		Rat po 15 mg/kg			Human po 40 mg		
		Observed	Predicted	Predicted/ Observed	Observed [43]	Predicted	Predicted/ Observed
C_max_	ng/mL	132 ± 35.3	69.3	0.53	58.8	36.4	0.63
t_1/2_	h	5.76 ± 1.45	2.96	0.51	2.80	3.44	1.23
AUC_inf_	ng·h/mL	408 ± 121	333	0.82	163	138	0.85

**Table 7:** Comparison of exposure data between observed and predicted values from PBPK models for saxagliptin and nicardipine.

Species	Dose (Route)	MRD	Pred/Obs Ratio		Reference
			C_max_	AUC_inf_	
Saxagliptin					
Rat	10 mg/kg (iv)	1.92	2.05	1.04	[19]
	5 mg/kg (po)	1.14	1.46	1.13	In-house data
	GMFE		1.73	1.08	
Human	2.5 mg (po)	1.32	1.22	0.96	[39]
	5 mg (po)	1.17	1.32	1.08	[40]
	5 mg (po)	1.60	1.15	0.73	[41]
	GMFE		1.23	1.16	
Nicardipine					
Rat	12 mg/kg (po)	1.69	0.91	1.33	[18]
	15 mg/kg (po)	2.08	0.68	0.85	In-house data
	GMFE		1.27	1.15	
Human	20 mg (po)	1.92	0.72	1.50	[42]
	40 mg (po)	1.66	0.63	0.86	[43]
	GMFE		1.48	1.33	

MRD: mean relative deviation, GMFE: geometric mean fold error.

**Table 8:** Comparison of plasma exposure parameters of saxagliptin following oral administration of without or with nicardipine.

Parameter	Rat						Human		
	Observed			Predicted by PBPK Model			Predicted by PBPK Model		
	SXG	SXG + NCD	DDI Ratio	SXG	SXG + NCD	DDI Ratio	SXG	SXG + NCD	DDI Ratio
C_max_ (ng/mL)	98.5 ± 15.1	281 ± 147	2.85	147 (130–154)	395 (377–407)	2.69	9.81 (7.36–12.9)	10.8 (8.15–14.2)	1.10
AUC_inf_ (ng·h/mL)	157 ± 23.1	408 ± 213	2.60	193 (152–222)	490 (381–577)	2.54	50.5 (35.2–78.2)	52.9 (36.8–82.3)	1.05

The values within parentheses represent the prediction interval, and the DDI ratios were calculated based on mean values.

## Data Availability

The data presented in this study are available in the article.

## Supplementary Materials

The following supporting information can be downloaded at: https://www.mdpi.com/article/10.3390/pharmaceutics16020280/s1. Supplementary material 1: Figure S1. Overall PBPK modeling scheme. Table S1. Organs and physiological parameters in PBPK models. Table S2. Partition coefficients (intracellular:plasma) of saxagliptin and nicardipine in PBPK models. Supplementary material 2: PK-Sim.file.
