# Supplementary material for: Physiologically Based Pharmacokinetic (PBPK) Modeling to Predict CYP3A-Mediated Drug Interaction between Saxagliptin and Nicardipine: Bridging Rat-to-Human Extrapolation
Source: Pharmaceutics. 2024 Feb 16;16(2):280. doi: 10.3390/pharmaceutics16020280 (PMC10892660; doi:10.3390/pharmaceutics16020280)
Supplement: Supplementary file 1 [file pharmaceutics-16-00280-s001.zip › Supplementary material S1.pdf]

Supplementary Material S1

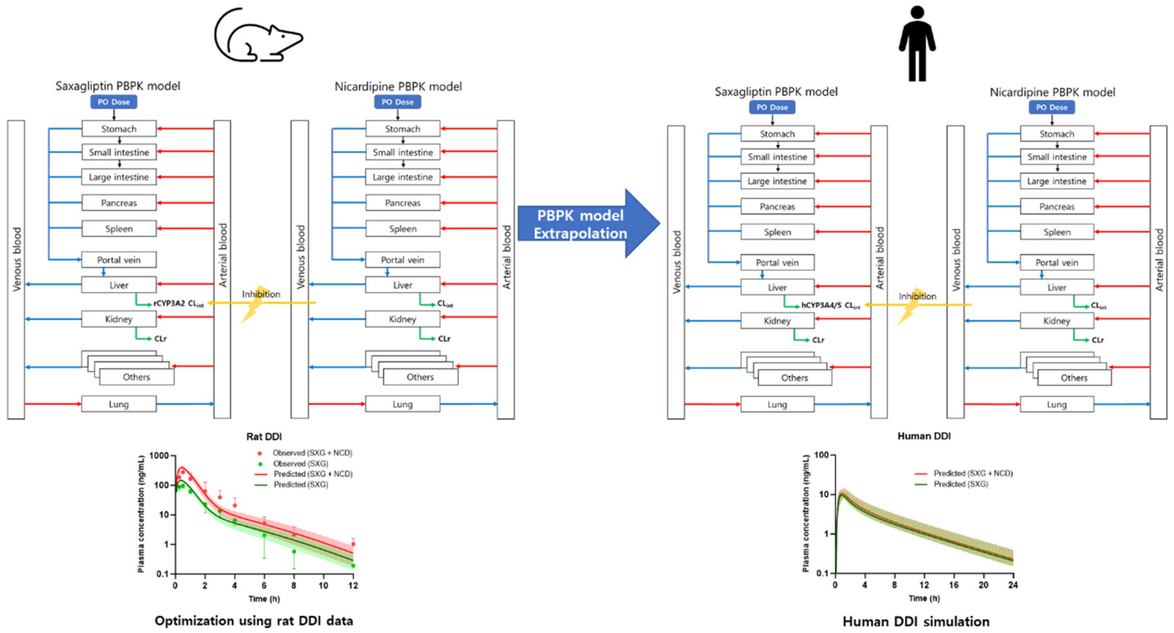

Figure S1. Overall PBPK modeling scheme

Table S1. Organs and physiological parameters in PBPK models

Species	Rat		Human	
Body weight (kg)	0.23		73	
Organ	Volume (L)	Blood flow rate (mL/min)	Volume (L)	Blood flow rate (mL/min)
Venous blood	0.00676		0.96	
Arterial blood	0.00294		0.42	
Bone	0.02	2.53	11.82	320
Brain	0.0017	1.33	1.51	780
Fat	0.01	0.4	14.65	320
Gonads	0.0025	0.48	0.04	3.25
Heart	0.0008	3.92	0.42	260
Kidney	0.0023	9.23	0.44	1330
Large intestine	0.00218	5.0	0.41	260
Liver	0.01	2.0	2.38	430
Lung	0.001	40	1.21	6090
Muscle	0.12	7.5	32.64	1120
Pancreas	0.0013	0.517	0.19	70
Portal vein	0.0015	9.78	1.04	1210
Skin	0.04	5.83	3.76	330
Small intestine	0.005	2.5	0.72	650
Spleen	0.0006	0.633	0.21	170
Stomach	0.0011	1.13	0.17	70

Table S2. Partition coefficients (intracellular:plasma) of saxagliptin and nicardipine in PBPK models.

**Species**	**Rat**		**Human**	
**Organ**	Saxagliptin	Nicardipine	Saxagliptin	Nicardipine
Bone	0.52	1740	0.63	109
Brain	0.71	712	0.87	44.5
Fat	0.42	5100	0.51	319
Gonads	0.68	214	0.83	13.4
Heart	0.66	661	0.80	41.3
Kidney	0.68	335	0.83	22.2
Large intestine	0.69	414	0.84	25.9
Liver	0.66	466	0.81	29.1
Lung	0.69	89.6	0.84	5.60
Muscle	0.69	108	0.85	6.74
Pancreas	0.63	528	0.77	33.0
Skin	0.58	678	0.71	42.4
Small intestine	0.69	414	0.84	25.9
Spleen	0.67	129	0.82	8.08
Stomach	0.69	414	0.84	25.9
